# Series of myocardial FDG uptake requiring considerations of myocardial abnormalities in FDG-PET/CT

**DOI:** 10.1007/s11604-021-01097-6

**Published:** 2021-01-31

**Authors:** Ryogo Minamimoto

**Affiliations:** Division of Nuclear Medicine, Department of Radiology, National Center for Global Health and Medicine, 1-21-1, Toyama, Shinjyuku-ku, Tokyo, 162-8655 Japan

**Keywords:** FDG, PET/CT, Myocardium, Physiological uptake, GLUT

## Abstract

Distinct from cardiac PET performed with preparation to control physiological FDG uptake in the myocardium, standard FDG-PET/CT performed with 4–6 h of fasting will show variation in myocardial FDG uptake. For this reason, important signs of myocardial and pericardial abnormality revealed by myocardial FDG uptake tend to be overlooked. However, recognition of possible underlying disease will support further patient management to avoid complications due to the disease. This review demonstrates the mechanism of FDG uptake in the myocardium, discusses the factors affecting uptake, and provides notable image findings that may suggest underlying disease.

## Introduction

For the better assessment of myocardial disease, long fasting or a high-fat, low-carbohydrate diet prior to 2-[18F]-fluoro-2-deoxy-d-glucose (FDG) positron emission tomography (PET) has been performed to suppress the physiological FDG uptake in the myocardium. However, standard PET/computed tomography (CT) performed with 4–6 h of fasting cannot control the physiological FDG uptake in the myocardium, thus variety of myocardial FDG uptake is observed. Even it is challenging to distinguish from physiological FDG uptake, important signs of myocardial and pericardial abnormality can be revealed by standard FDG-PET/CT. This review presents the mechanism of FDG uptake in the myocardium, discusses the factors affecting uptake, and provides notable image findings that may suggest underlying disease.

## Mechanism of FDG uptake in the myocardium

The energy requirements of the myocardium are supplied mainly by fatty acids (FA), carbohydrates, and ketone bodies [1]. The glucose metabolism status of the myocardium changes according to the available substrate and myocardial function and perfusion. When plasma glucose and insulin levels rise, glucose transporters (GLUT) in the myocardium (GLUT-1 and GLUT-4) increase the myocardial glucose intake. In the fasting state, plasma insulin levels fall and cardiac energy requirements are supplied mainly by FA following the reduction in oxidative glucose metabolism obtained from carbohydrates [2]. To reduce physiological FDG uptake in the myocardium, 18–24 h fasting is required, because the human myocardium preferentially utilizes energy derived from free fatty acids rather than from glucose during the fasting state in aerobic conditions. Standard FDG-PET/CT imaging protocols generally require at least 4–6 h of fasting before the examination. Accordingly, the metabolic shift in the myocardium is not completely accomplished, and a variety of myocardial physiological uptake patterns are present in standard FDG-PET/CT [3].

## Factors affecting myocardial FDG uptake

Major factors affecting myocardial glucose metabolism include sex differences, aging, obesity, and diabetic mellitus. Compared with the male myocardium, the female myocardium requires more oxygen and FA, and less glucose. Metabolic change also occurs in pathological states such as obesity, diabetic mellitus, and nonischemic cardiomyopathy [4]. Estrogen upregulates nitric oxide synthesis, leading to a reduction in GLUT-4 translocation to the cell surface [5, 6]. The higher percentage of body fat in females than males leads to higher plasma FA levels and incorporation of FA to the heart in females [4, 7]. Structural changes in the myocardium such as increased myocyte size and fibrosis show progression with age. The contribution of FA oxidation to myocardial metabolism decreases with age for multifactorial reasons related to mitochondrial status, free radical injury, a decline in peroxisome proliferator-activated receptor alpha (PPARα) activity, and increased pyruvate oxidation [8–12].

An increase in body mass index leads to increased myocardial FA metabolism. In females, the dependence on myocardial FA metabolism increases with worsening insulin resistance, with little change in myocardial glucose metabolism; and myocardial volume oxygen consumption is greater in obese females than in obese males. In contrast, obese males have greater impairment of myocardial glucose metabolism than obese females at the same level of plasma insulin, suggesting greater myocardial insulin resistance [13].

Systemic insulin resistance induces an increase in plasma FA delivery, leading to stimulation of FA intake to the myocardium. The increased FA metabolism and decreased glucose use that occurs in diabetic mellitus is related to the proliferator-activated receptor coactivator 1 alfa signaling network and protein kinase C [14].

Blood glucose level does not directly correlate with physiological myocardial FDG uptake [15]. Renal failure have no influence on physiological myocardial FDG uptake 16]. Physiological FDG uptake in the myocardium varies among patients and even in the same patient at different time points during scanning, which appears to be related to the patient’s metabolic and hormonal status at the time of scanning [17].

Myocardial FDG uptake can be influenced by bezafibrate, levothyroxine, thiazolidinedione, and benzodiazepine [18, 19]. Bezafibrate reduces serum triglyceride levels by altering lipoprotein metabolism [20, 21], and also lowers blood glucose, HbA1C, and insulin resistance in attenuating the progression of diabetic mellitus type 2. The expression of glucose transporters and activity of phosphofructokinase-1 is decreased in hypothyroid rats [22, 23]. The thyroid hormone levothyroxine can stimulate glucose transport and glycolysis by upregulating GLUT-4 transcription [24], and decreased myocardial FDG uptake has been reported in patients prescribed levothyroxine [19]. Thiazolidinediones are ligands for PPARγ, which regulates adipocyte differentiation and glucose homeostasis by improving insulin sensitivity and secretion, glucose tolerance, and adipocytokines in patients with diabetic mellitus type 2 [25, 26]. This mechanism might be associated with reduced FDG uptake in the myocardium. Benzodiazepine receptors are present in the central nervous system and in peripheral tissue, including the myocardium [27], but the detailed mechanism of increased FDG uptake in the myocardium remains unknown.

## Myocardial uptake variability

**Fig. 1:**
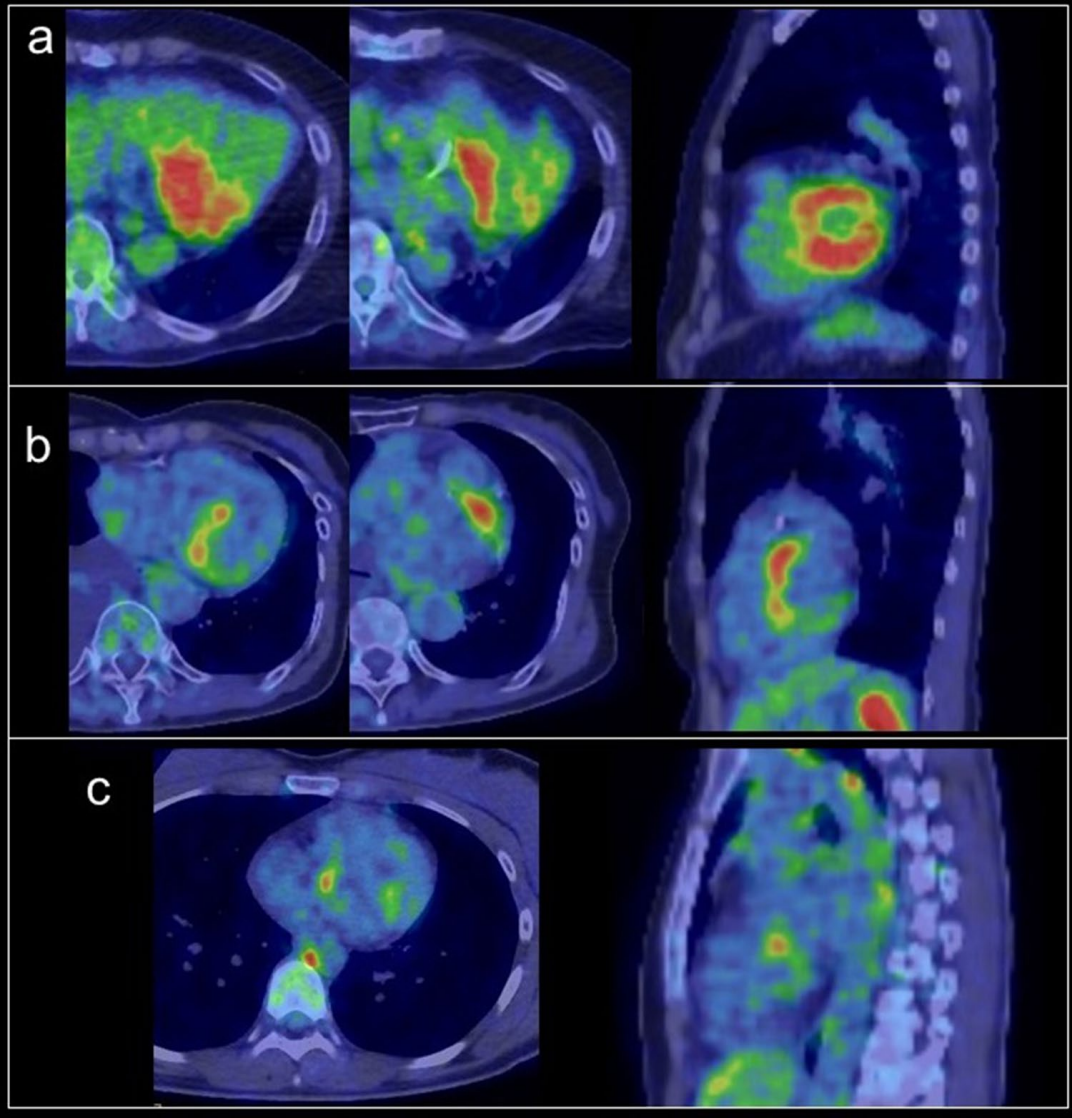
Predominant regional FDG uptake in the base of the myocardium. **a** ring pattern, **b** over-half-ring pattern, **c** spot pattern

The physiological FDG uptake pattern in the myocardium is classified as focal, regional, diffuse type, or none [15, 18, 28]. As it is not dependent on age, glucose level, weight, or FDG dose, the uptake pattern will be poorly-reproducible in the following PET examination. Regional FDG activity is generally lower in the septum and anterior left ventricular (LV) wall than in the lateral and posterior walls [3, 17, 29, 30]. An experimental animal study found that wall stresses were highest in the anterior, lateral, and anterior papillary regions of the myocardium, which suggests that increased myocardial wall stress leads to increased metabolic demand [31]. Predominant regional FDG uptake in the base of the myocardium is also a common physiologic uptake pattern [28], occurring as ring pattern, over-half-ring pattern, and spot pattern (including focal in diffuse FDG uptake) [32] (Fig. 1).

**Fig. 2:**
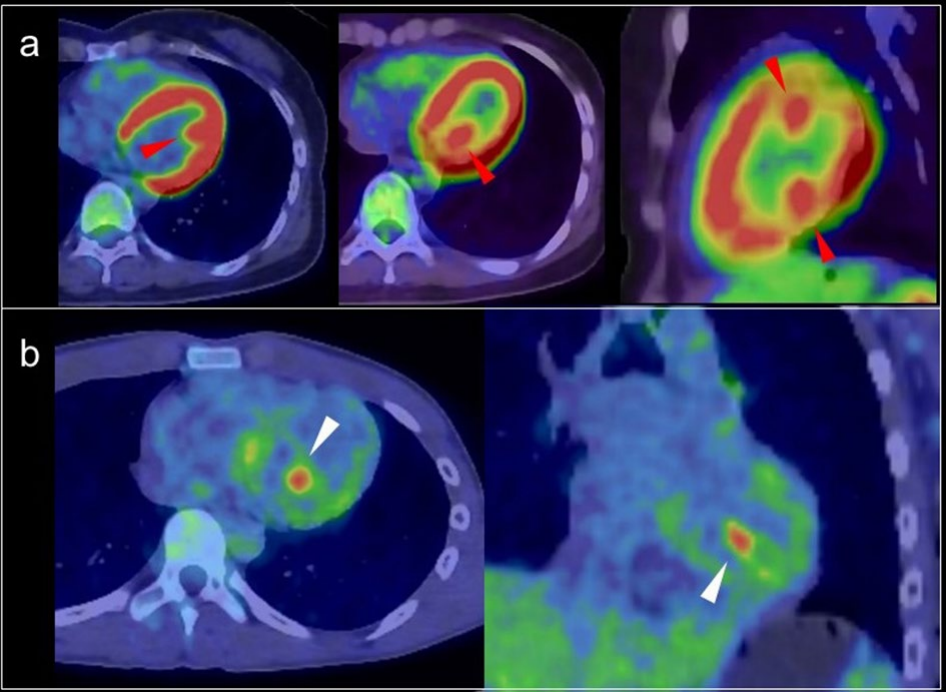
Papillary muscles. **a**; FDG uptake at left ventricle papillary muscles in the anterolateral locations and infero-posterior locations (red arrowheads), **b** Isolated FDG uptake in the left ventricle (white arrowheads) was suspected thrombus but no evidence was found with cardiac ultrasonography. The FDG uptake was continued from pupillary muscles with slight FDG uptake, finally the FDG uptake was regarded as physiological uptake in pupillary muscles and/or chordae tendineae

A focal FDG uptake pattern is sometimes confirmed as accumulation in the LV papillary muscles in their anterolateral and infero-posterior locations, where it is commonly observed in combination with FDG uptake in the adjacent myocardium (Fig. 2). In contrast, isolated FDG uptake in the papillary muscle is rare, and may suggest thrombus or neoplasm [33].

The basal segment FDG activity was present in 57% of all subjects, comprising increased basal-lateral (54%), postero-basal (32%), basal-anterior-basal (15%), and basal (15%) septum patterns [18]. Nose et al. found that the FDG uptake pattern in subjects without heart disease was none in 28%, diffuse in 34%, focal/diffuse in 21%, and focal in 18% [34]. Focal uptake was seen most commonly in the basal wall. Uptake pattern was not influenced by age, blood glucose level, body weight, or injected dose of FDG. Based on the coronary branch territories, mean uptake was higher in the left circumflex coronary artery than right coronary artery (RCA) territories, and lower in the left coronary artery than RCA territories. A basal–apical gradient analysis showed that uptake was significantly higher in the middle territories than in the proximal and distal territories [29].

## Normal variant finding

### Crista terminalis

**Fig. 3:**
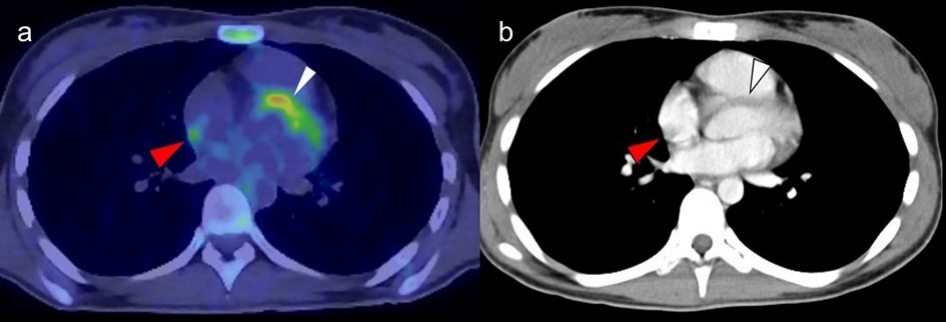
Crista terminalis. Mild focal FDG uptake was observed in the right atrium (arrowhead) (**a**), which corresponded to the crista terminalis confirmed by contrast enhanced CT (**b**). In addition, linear FDG uptake correspond to the fat tissue suggested lipomatous hypertrophy (white arrowhead)

The crista terminalis is a smooth, crescent-shaped muscle band that separates the right atrium (RA) from the right atrial appendage [35]. It originates from regression of the septum spurium as the sinus venosus is incorporated into the right atrial wall. Its thickness varies widely among adults (3–6 mm) [36]. The sinoatrial node lies on the upper part of the RA, and its identification is important in cardiac electrophysiology examinations [37]. Because this muscular band occasionally shows increased FDG uptake, it should not be misinterpreted as myocardial tumor, thrombus, or focal pericardial metastases [38, 39] (Fig. 3).

### Lipomatous hypertrophy of the interatrial septum

**Fig. 4:**
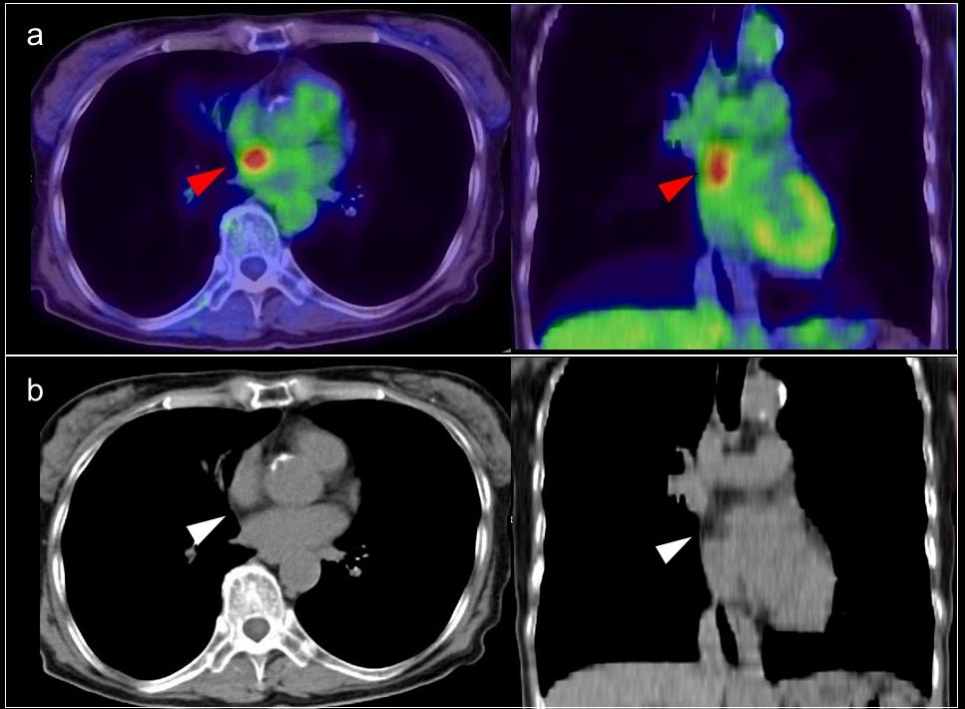
FDG uptake in lipomatous hypertrophy of the interatrial septum. Focal FDG uptake between left and right atrium (**a** red arrowheads), which corresponded to lipomatous hypertrophy of the interatrial septum (**b** white arrow heads)

Lipomatous hypertrophy of the interatrial septum (LHIS) is a benign condition with prevalence of 1–8% that is associated with aging, sex (female), and obesity [40]. LHIS has a dumbbell-shaped appearance with significant thickening of the interatrial septum, with 20 mm or more showing with fat density on CT that extends to the atrial wall but rarely to the interventricular septum, and sparing of the fossa ovalis [41, 42]. It is composed of mature adipocytes and fetal fat cells or brown fat, and FDG uptake is thought to be dependent on the volume of brown adipose tissue (BAT) [43, 44] (Fig. 4). BAT is an adipose organ that functions to maintain core temperature in small mammals and in newborn humans [45]; however, FDG uptake representing the metabolic activity of BAT proves its existence also in adult humans [46]. Most subjects with LHIS are asymptomatic, but it is known to be associated with supraventricular arrhythmias, syncope, and sudden death [47, 48]. A previous study found that in 82% of these patients, focal increased FDG uptake corresponding to the regions of LHIS [44]. Brown fat is sometimes apparent as focal areas of FDG uptake that are localized to the mediastinum around the pericardium and epicardium in up to 1–2% of patients [49], which can be mistaken as mediastinal adenopathy or malignant pericardial infiltration.

## Myocardial finding

### FDG uptake in the atrial wall

**Fig. 5:**
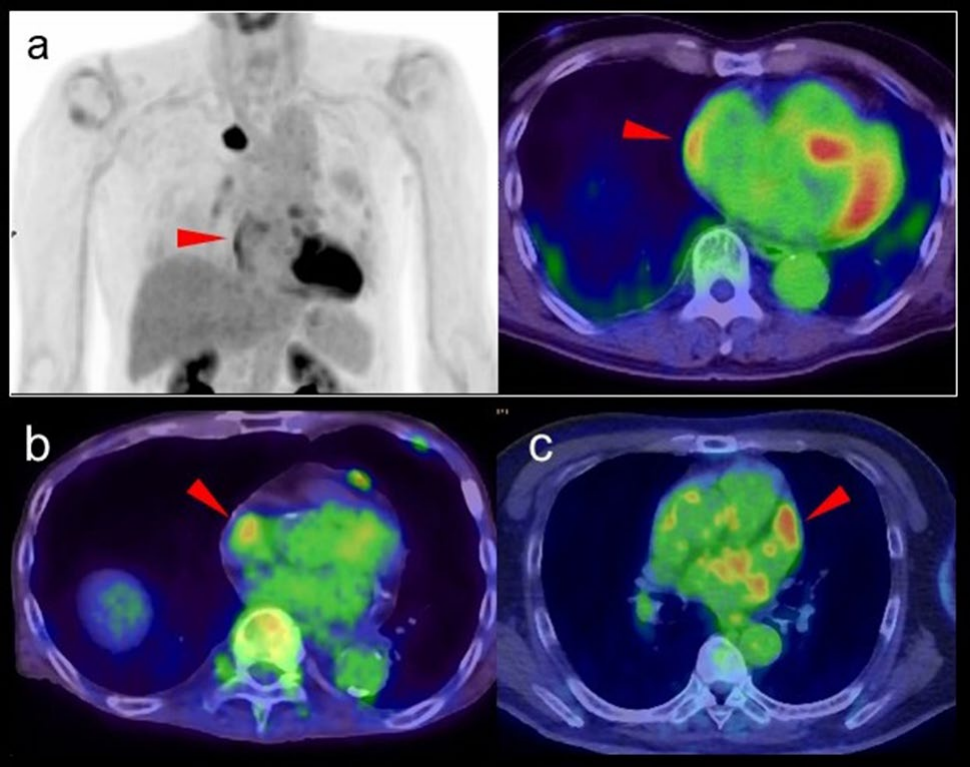
FDG uptake pattern associated with atrial fibrillation. Increased FDG activity along with the atrial wall (**a** red arrowheads), FDG uptake at right appendage (**b** arrowhead) and at left appendage (**c** arrowhead) are all associated with atrial fibrillation

FDG uptake is not usually seen in the atrial wall because of its much lower energy consumption compared with the ventricles. Increased FDG activity along with or localized in the atrial wall (including the atrial appendage) is associated with atrial fibrillation (AF), even in the case of cardiac chamber enlargement. However, not all patients with AF show increased FDG activity in the atrial wall [50, 51] (Fig. 5).

Xie et al. proposed that activity in the epicardial adipose tissue was an independent factor predicting increased activity in the atrium [50]. Joseph et al. showed a relationship between higher hematopoietic tissue activation and the incidence of AF [52].

The incidence of AF was significant associated with SUVmax of FDG in the RA and volume of the left atrium (LA). Moreover, a pathological investigation reported infiltration of extravascular macrophages and lymphocytes in regions with FDG uptake in the atrium [53]. Sinigaglia et al. reported a strong association of FDG uptake in the right and LA with increased prevalence of stroke in patients with AF [54].

### Increased FDG uptake in the right ventricle

**Fig. 6:**
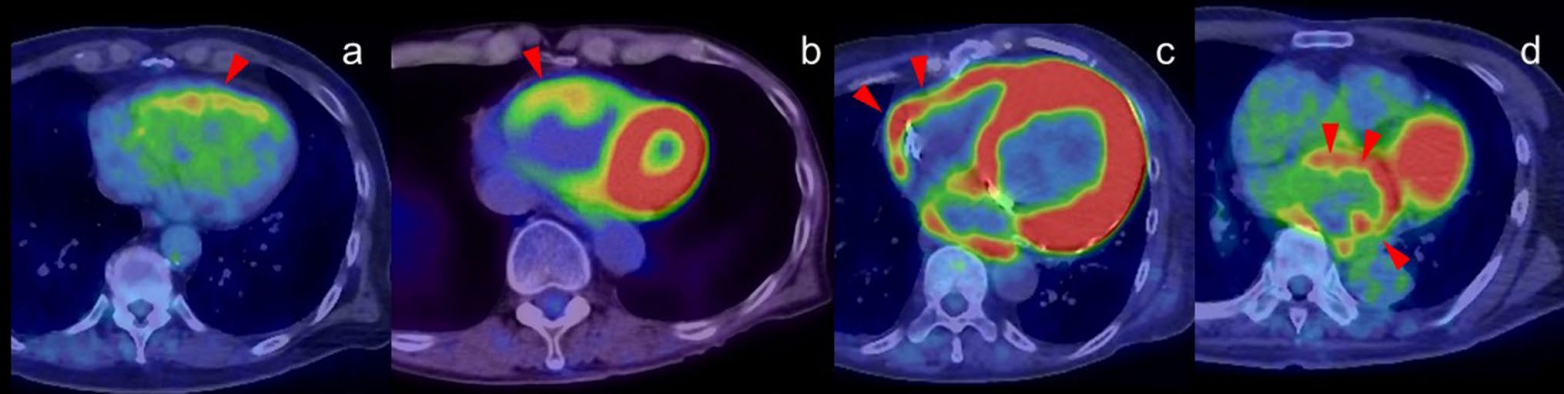
FDG uptake in right ventricle. **a** Diffuse FDG uptake in the enlarged right ventricles (arrowhead) caused by increased ventricular pressure in patient with long history of emphysema. **b**, **c** Diffuse FDG uptake in the enlarged right ventricles (arrowhead) caused by left ventricle hypertrophy. **d** Diffuse FDG uptake in the enlarged left atrium suggested increased atrial pressure

Diffuse FDG uptake in the right ventricle (RV) has been identified in the setting of increased ventricular pressure or overload in patients with pulmonary hypertension (Fig. 6). Enlargement of the LV and RV with a diffuse increase in FDG myocardial activity is seen in association with systemic hypertension, valvular heart disease, and other myopathies [55–58] (Fig. 6). When atrial FDG uptake is confirmed in an enlarged atrium accompanied by diffuse uptake in the RV, atrial FDG uptake can also be considered to be caused by increased pressure or overload [58], and the increased level is a marker of poorer prognosis [59]. In our experience, FDG uptake in the RV is more frequently seen in healthy young adults, but the mechanism is unclear.

### Myocardial hypertrophy

**Fig. 7:**
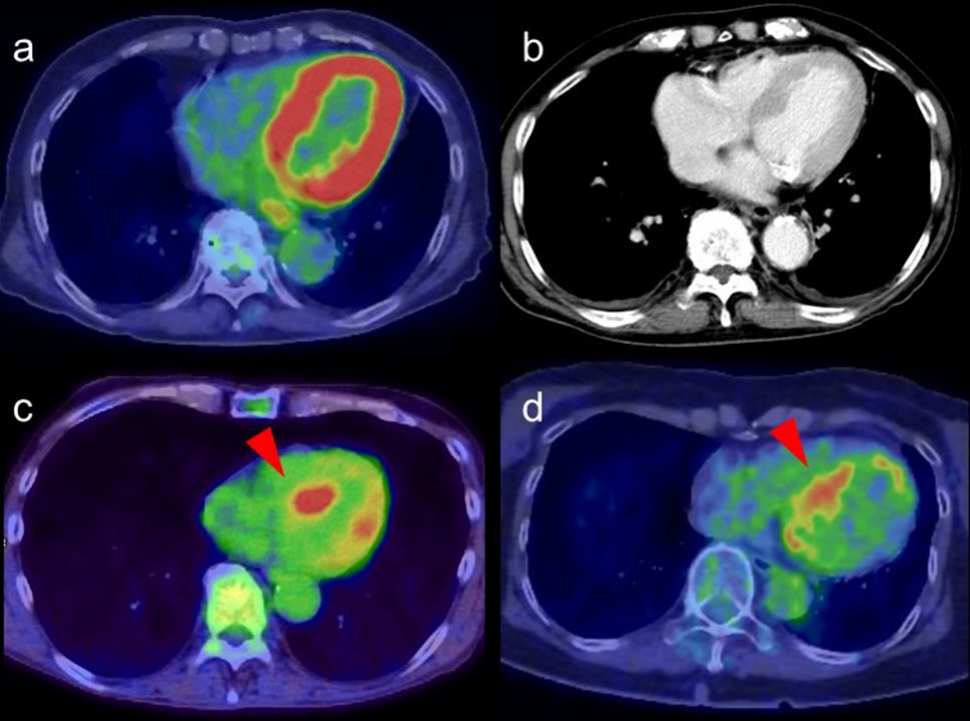
FDG uptake in left ventricular hypertrophy. Intense FDG uptake was confirmed along with the left ventricular wall (**a**). Contrast enhanced CT suggested thickening of left ventricular wall suggesting left ventricular hypertrophy (**b**). Myocardial FDG uptake features are shown with hypertrophic obstructive cardiomyopathy (**c**) and with hypertrophic nonobstructive cardiomyopathy (**d**) (red arrowheads)

The expression of beta-oxidation enzymes is decreased as myocardial hypertrophy progresses, leading to decreased myocardial FA as glucose use increases in the myocardium. Therefore, myocardial FDG uptake increases in left ventricular hypertrophy (LVH), and the uptake is reported to be more prominent in hypertrophic obstructive cardiomyopathy than in hypertrophic nonobstructive cardiomyopathy [60] (Fig. 7). In contrast, FDG uptake in the myocardium can decrease in patients with LVH if the metabolic remodeling proceeds as structural remodeling in the form of LVH. The decrease in diastolic function leads to a decline in FDG uptake. The reduction of FDG uptake in progressive LVH that is caused by underlying tissue characterization such as fibrosis indicates the risk of myocardium progressing to heart failure [61–63].

### Focal FDG uptake in the left ventricular apex

**Fig. 8:**
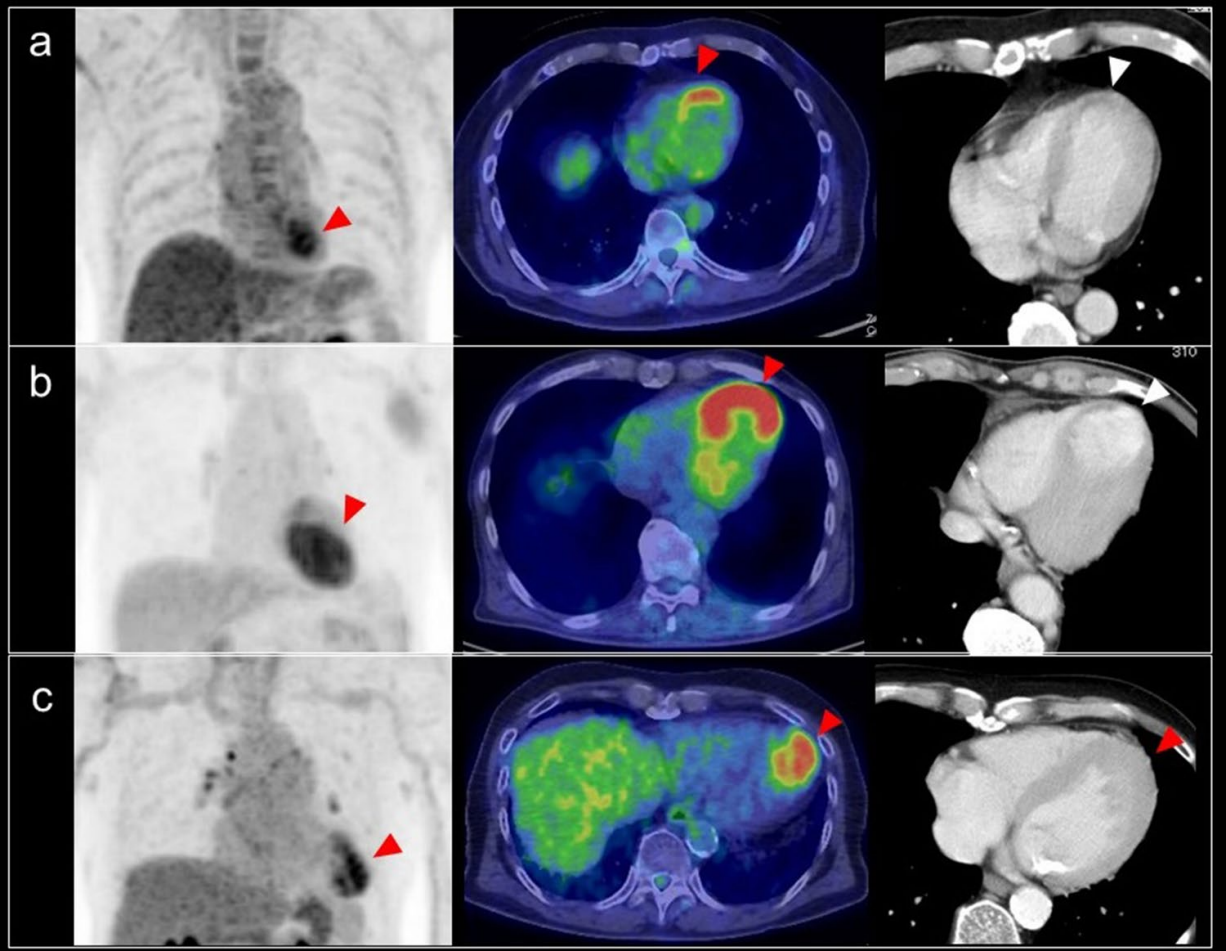
Focal FDG uptake in the left ventricular apex. **a** focal FDG uptake in the left ventricular (LV) apex (red arrowheads) matched the liner unenhanced CT area suggesting chronic ischemic change of the apex (white arrowhead). **b** intense focal FDG uptake in the LV apex (red arrowheads) was matched to the LV aneurysm (white arrowhead). **c** apical hypertrophic cardiomyopathy can also show focal FDG uptake in the LV apex (red arrowheads)

Focal FDG uptake in the LV apex is a rare finding, occurring up to 0.6% of oncologic FDG-PET/CT scans [64, 65]. A previous study reported that 55% of subjects with focal FDG uptake in the LV apex had coronary artery stenosis or a history of treatment for coronary disease (Fig. 8), and that 10% had apical hypertrophic cardiomyopathy [64]. Takanami et al. reported the ‘focal high’ and ‘focal defect on diffuse high’ pattern of myocardial FDG uptake in the LV apex were correlated with abnormal myocardial perfusion imaging (MPI) [66]. Haider et al. observed focal myocardial FDG uptake (not limited to the apex) in patients with myocardial abnormalities such as abnormal perfusion, impaired LV ejection fraction (LVEF), myocardial ischemia, and scarring. In addition, focal myocardial FDG uptake has been identified as a strong predictor of abnormal myocardial function/perfusion and as an independent predictor of ongoing ischemia and myocardial scarring [67].

Increased FDG uptake can persist for 48 h after stress-induced myocardial ischemia in stable coronary artery disease [68, 69]. Dou et al. reported that ‘focal’ or ‘focal on diffuse’ uptake was seen in 86% of patients with unstable angina but in only 8% of patients without unstable angina [70]. However, it has also been shown that abnormal MPI is possible even in the case of low FDG uptake (44%) and basal ring uptake (43%), although less commonly for basal ring pattern (11%) [66]. Akikawa et al. reported an incidental finding of focal myocardial FDG uptake, indicating asymptomatic coronary artery disease, which disappeared following successful percutaneous coronary intervention [71].

Apical hypertrophic cardiomyopathy is another possible cause of focal FDG uptake in the LV apex (Fig. 8). Its characteristic appearance is an unusual pattern of hypertrophic cardiomyopathy with wall thickening limited to the apex of the LV. Focal FDG uptake in the LV apex shows a tendency to occur more frequently in patients with the echocardiography findings of apical wall thickness ≥ 15 mm and asynergy in the apex. FDG uptake in the LV apex is seen in patients with decreased coronary flow reserve, indicating an association with microvascular dysfunction [65].

### Localized FDG uptake in the left ventricle

**Fig. 9:**
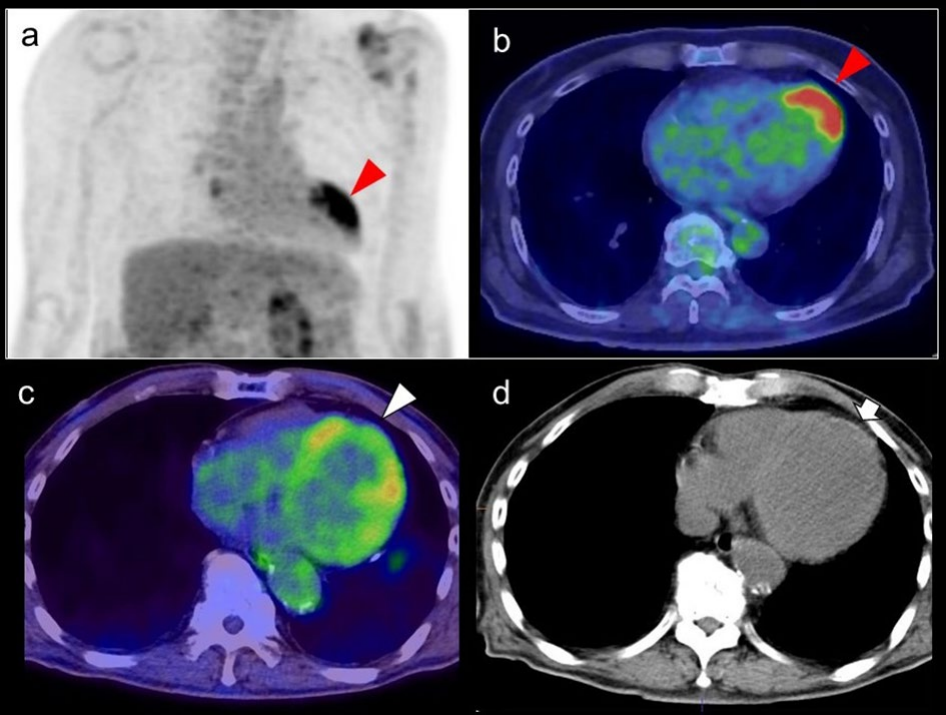
Myocardial ischemic change. Focal FDG uptake in the anterior of myocardium (**a**, **b** red arrowheads) had a possibility of ischemic change, which is caused by metabolic switch from fatty acid to glucose use. Whereas defect of FDG uptake at apex (**c** white arrowhead) with slight calcification on CT (**d** white arrow) suggest chronic myocardial infarction

**Fig. 10:**
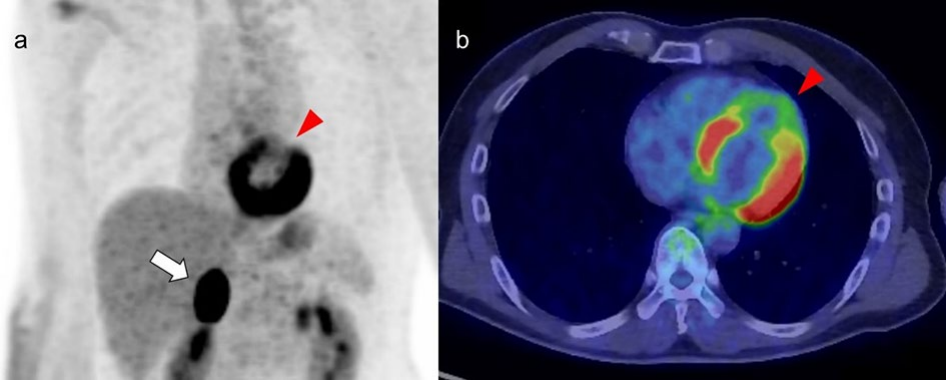
Catecholamine induced myocarditis. Focal FDG uptake is seen in the pheochromocytoma arisen at right adrenal grand (**a** white arrow). Decreased patty FDG uptake in the anterior wall to apex of left ventricle (**a**, **b** red arrowheads) indicated catecholamine induced myocarditis

A shift from fatty acid metabolism to glucose utilization in the myocardium occurs in ischemia. Based on this mechanism, increased FDG uptake in the LV wall can be recognized in the case of chronic ischemia, termed hibernating myocardium [72]. Localized FDG uptake is more likely to indicate an ischemic state if the uptake corresponds to the coronary artery distribution (Fig. 9). Physiological FDG uptake in the posterolateral wall can mimic ischemia in the left circumflex coronary artery [18], whereas decreased or absent FDG uptake indicates myocardial scarring with irreversible functional damage [73] (Fig. 10). A reduction of septal FDG uptake has also been observed in left bundle branch block (LBBB) [74].

Decreased FDG uptake also occurs during the time course of Takotsubo cardiomyopathy [75] (Fig. 10). In the acute and subacute phases, FDG defects are demonstrated in the hypo-contractile LV segments even if perfusion is only slightly reduced. Later, there is rapid normalization of myocardial perfusion, whereas recovery of glucose metabolism is delayed. Although stress-induced catecholamine overproduction and myocardial response are the most important mechanisms [76], coronary microvascular dysfunction may cause transient perfusion abnormalities, whereas excessive catecholamine exposure induces toxic effects in cardiomyocytes, including abnormalities such as glucose metabolism disorder [77]. Representative causes of excessive catecholamine are pheochromocytoma, stress, medications, and ingestion of exogenous substances [78].

### History of chemotherapy or radiation therapy

**Fig. 11:**
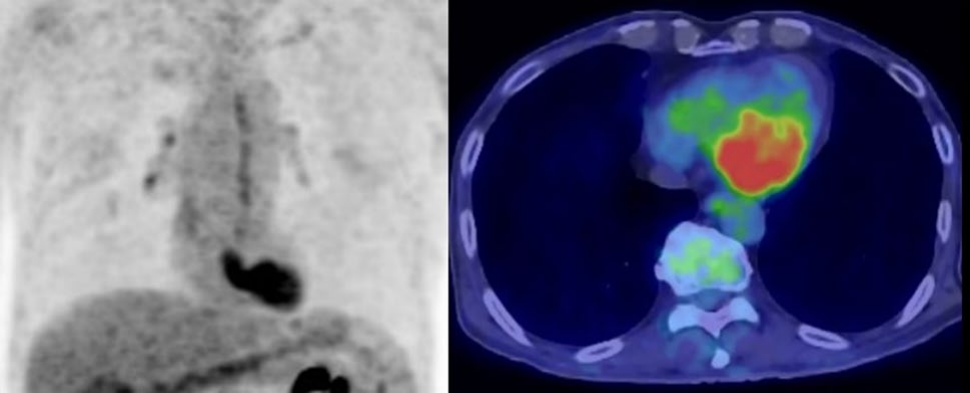
Cardiotoxicity induced by Adriamycin. Myocardial dysfunction was occurred in the patient with diffuse large B cell lymphoma treated by R-CHOP. Increased focal FDG uptake in the myocardium was confirmed during the therapy. The patient was diagnosed with cardiotoxicity induced by adriamycin

Diffuse and increased FDG accumulation in the myocardium is a possible early sign of cardiotoxicity, which has been reported in patients treated with anthracyclines such as doxorubicin and adriamycin [79–83], tyrosine kinase inhibitors [84], and trastuzumab [83, 85] (Fig. 11). Increased FDG uptake in the myocardium during chemotherapy including doxorubicin is associated with a decline in LVEF [83]. The cardiac FDG uptake has been shown to progressively increase during therapy administration and remain elevated for at least 1 year [82]. Doxorubicin inhibits fatty acid oxidation and mitochondrial oxidative phosphorylation [86], leading to Pasteur effect and further increasing glucose consumption. Because the increase in glucose consumption is transient and disappears rapidly after drug removal [87], Bauckneht et al. suggested the capability of FDG for selectively tracking the early endoplasmic reticulum pentose phosphate pathway (PPP) response to oxidative stress [81].

Increased myocardial FDG uptake has been reported following radiation therapy for esophageal cancer [88–90] and lung cancer [91]. In this setting, FDG uptake has the feature of unusual sharp borders that correlate with the area of heart involved in the radiation therapy planning field, rather than following a typical coronary artery distribution [92]. Localized myocarditis with increased FDG activity generally occur at radiation doses > 35 Gy [88]. In an animal study, increased FDG uptake caused by radiation-induced myocardial damage appeared to reflect microvascular damage and mitochondrial injury [93].

### Possibility of endocarditis

Endocarditis is a life-threatening inflammation of the inner lining of the heart (the endocardium), and infectious endocarditis (IE) on both native valve and prosthetic valve (PVE), and cardiovascular implantable electronic device (CIED) infections are representative diseases. Meta-analyses showed that the overall pooled sensitivity of FDG PET/CT for the indication of IE and PVE is 61% and 73%, respectively [94]. The European Society of Cardiology (ESC) guidelines recommend using FDG-PET/CT for the diagnosis of endocarditis, expecting the reduction in the rate of misdiagnosed IE, classified in the Possible IE category with the Duke criteria [95, 96]. In addition to the detection of endocarditis, whole-body PET/CT images can contribute to peripheral embolization and/or metastatic infection [97]. Focal FDG uptake in the valve and devices indeed have possibly of the active endocarditis; however, physiological myocardial FDG uptake and overcorrection artifacts caused by metal artifacts may be a misinterpretation of the disease [98]. Non-specific FDG uptake in the immediate postoperative period can mimic the disease, thus the ESC guidelines stated that FDG PET result is not reliable within 3 months from prosthetic valve implantation [95].

### Incidence of cardiac sarcoidosis

**Fig. 12:**
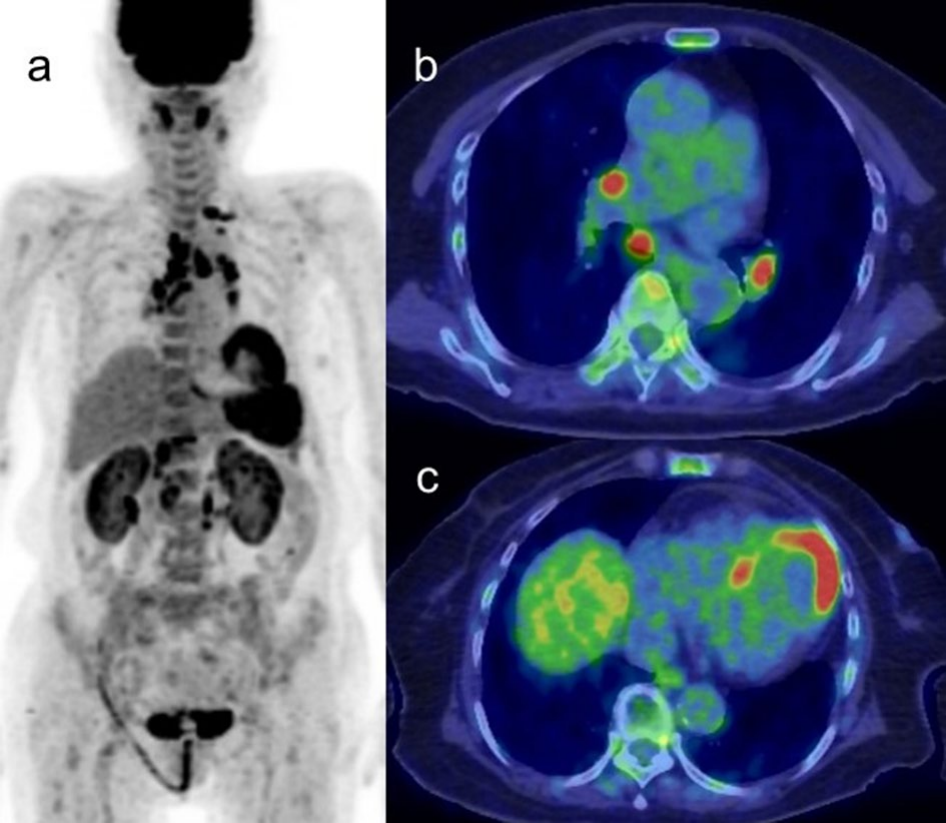
Cardiac sarcoidosis. Cardiac sarcoidosis in 70-year-old female. The patient showed multiple lymph node swelling on CT and suspected malignant lymphoma. FDG-PET/CT showed FDG uptake in the lymph nodes, and finally diagnosed with sarcoidosis. FDG-PET/CT presented intense FDG uptake in the left ventricular wall, finally diagnosed with cardiac involvement of sarcoidosis

Sarcoidosis is a systemic disorder of unknown etiology, characterized by the presence of noncaseating granulomas. Cardiac involvement occurs in only about 5% of those with systemic sarcoidosis, but leads to an adverse prognosis that causes approximately 25% of deaths from sarcoidosis. According to pathologic assessment, the presence of occult cardiac granulomas is around 20–58% in the patient with sarcoidosis, which is much higher than might be expected [99–101]. The uptake patterns of patchy nonhomogeneous FDG uptake or focal-on-diffuse FDG uptake under the suppression of physiological myocardial uptake indicate the existence of active cardiac sarcoidosis (CS), and perfusion imaging can increase the accuracy of diagnosis [99]. The presence of clinical symptoms related to CS and positive findings outside the myocardium suggestive of sarcoidosis can improve the diagnostic accuracy (Fig. 12).

The prevalence of isolated cardiac sarcoidosis (ICS), or sarcoidosis that involves only the heart, occurs in 3.1–25% of cases of CS [102]. Therefore, CS is generally an extra cardiac sarcoid lesion that can be differentiated from cardiac sarcoidosis by the finding of patchy nonhomogeneous FDG uptake or focal-on-diffuse FDG uptake.

### Possibility of cardiac tumor

The vast majority of cardiac tumors are secondary tumors caused by metastatic spread or by direct neoplastic invasion. Secondary tumors are 20–40 times more common than primary cardiac tumors [103–106]. Up to 12% of oncology patients have metastases to the heart or pericardium at autopsy, although most remain clinically silent [107]. Melanoma, breast, lung, and esophageal carcinomas are the most common to metastasize to the heart.

**Fig. 13:**
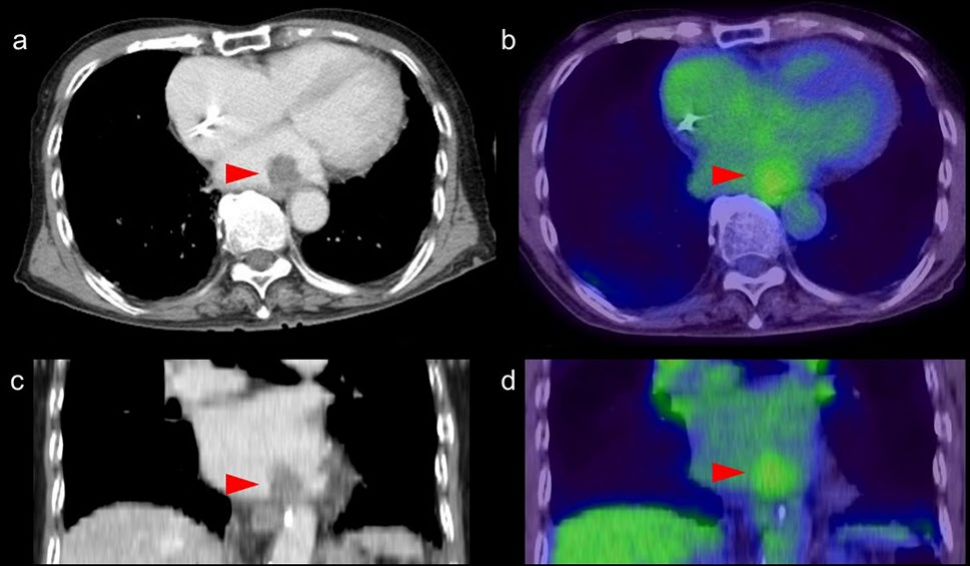
Myxoma arisen in left atrium. Contrast enhanced CT imaging demonstrated mass lesion in left atrium, which showed low FDG uptake

Primary cardiac tumors occur with a reported frequency of 0.0017–0.33% of the population, among which 75–90% are benign, and of which cardiac myxomas account for almost one half [108–110]. Cardiac myxomas have the typical appearance of a polypoid left intracavitary mass rather than a right atrial mass originating from the interatrial septum, and characteristically display either no significant or mildly elevated FDG uptake [18, 28, 108, 111] (Fig. 13). Common complications of myxoma such as valvular obstruction and embolism, as well as more generalized symptoms, may help identifying the tumor.

Cardiac lipoma is well-circumscribed spherical or elliptical mass composed of homogeneous yellow fat and located most commonly in the epicardium, but can be distributed in other areas of the heart such as the endocardium and myocardium [112]. Lipomas show no intense FDG uptake. Other possible benign tumors are fibroma and rhabdomyoma, which are most common in children.

Approximately 5–25% of primary cardiac tumors are malignant [107, 108], most of which show intense FDG uptake and SUVmax of 3.5–5 can be optimal cut off value for distinguishing malignant from benign cardiac tumor [113, 114]. Cardiac sarcoma is the most common primary malignant tumor of the myocardium (~ 65% of malignant primary cardiac tumors), of which angiosarcoma accounts for more than 33% [106, 107, 115]. Angiosarcoma is a highly aggressive and infiltrative tumor, located most commonly in the RA and pericardium, and shows high FDG uptake [113]. Liposarcoma [116] and myeloid sarcoma [117] have been reported as FDG-avid lesions arising in the myocardium, whereas osteosarcoma [118] is FDG-avid lesions located in the pericardium.

**Fig. 14:**
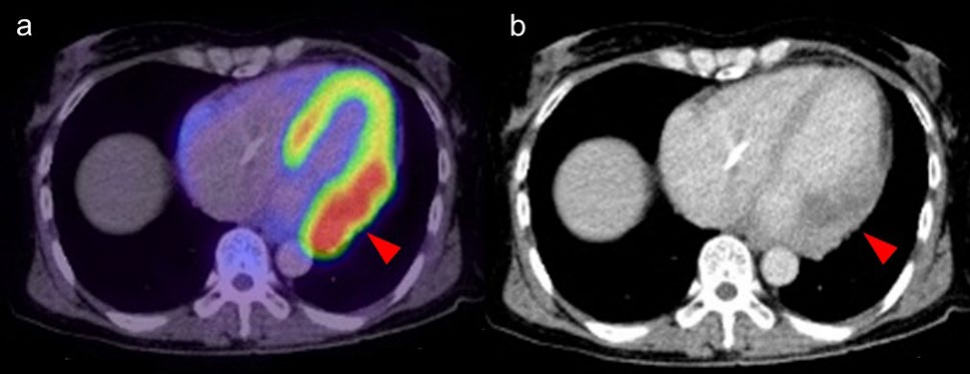
Primary cardiac lymphoma. Intense FDG uptake at left lateral wall was pathologically diagnosed diffuse large B cell lymphoma

Primary cardiac lymphomas (PCL) are an uncommon malignancy, accounting for 1.3% of primary cardiac tumors, rather it is much higher incidence as extensive lesion from the other [119]. PCL are generally of the aggressive B-cell lymphoma type, and most commonly involve the right side of the heart, particularly the right atrium. Cardiac lymphomas are characterized by significant FDG uptake due to their aggressive nature [120, 121] (Fig. 14). No intraluminal involvement regardless of surroundings of coronary artery may help the diagnosis of cardiac lymphomas. The other possible FDG-avid malignant lesion is leukemia such as extramedullary AML extending to the myocardium and pericardium, which used to be a clinically undetectable extramedullary lesion [122]. The value of FDG-PET/CT is its ability to differentiate between benign and malignant tumors and to optimize the biopsy location.

### Possibility of cardiac amyloidosis

**Fig.15:**
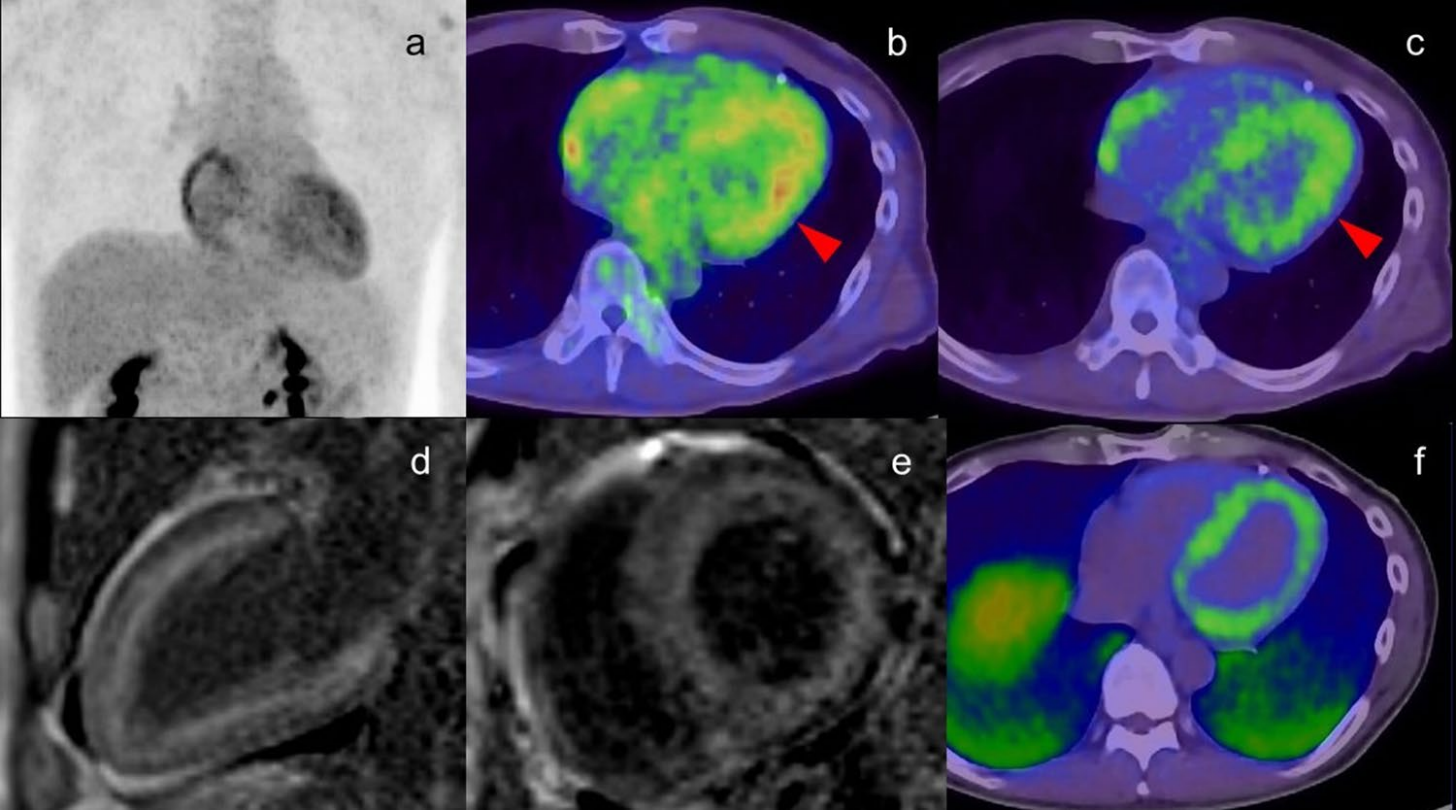
Cardiac amyloidosis. Moderate FDG uptake was confirmed in left ventricle wall in the patient with AL amyloidosis (**a**, **b** red arrowhead). Different color scale of FDG PET/CT imaging demonstrated that FDG uptake existed in inner layer of left ventricle wall, which were matched to the area with high intensity in PSIR MRI image (**d**, **e**) and the uptake in PiB PET/CT imaging (**f**) suggesting the amyloid deposit. The FDG uptake in the right atrium was cause by overload pressure of right atrium

High and diffuse myocardial FDG uptake has been reported in patients with immunoglobulin-derived light chain (AL) amyloidosis deposits in the myocardium [123, 124]. Due to the small number of reports and similarity to physiological uptake, the typical uptake pattern in cardiac amyloidosis is not obvious. In addition, it is uncertain whether the FDG uptake in any pathological type of amyloidosis can be confirmed. In localized AL amyloidosis, giant cells play a role in the production of amyloid, whereas giant cells are thought to be unnecessary for the formation and deposition of amyloid in systemic AL, amyloid A amyloidosis and transthyretin amyloidosis. The presence of giant cells and other inflammatory cells is thought to lead to increased FDG uptake [125, 126]; therefore, intense FDG uptake would be more likely in localized AL amyloidosis than in systematic amyloidosis [127] (Fig. 15).

Plasma cell malignancies such as multiple myeloma are characterized by clonal expansion of terminally differentiated B lymphoid cells, resulting in the production of monoclonal immunoglobulins or fragments [124]. FDG-PET/CT is a reliable method for the detection of active multiple myeloma, in which 12–15% of patients develop clinical amyloidosis during the course of the disease, and subclinical amyloid deposits are found in multiple organs, including the myocardium, in up to 30% of myeloma patients [128]. Amyloid cardiac involvement can cause life-threatening complications; therefore, recognition of AL amyloidosis in patients with multiple myeloma is crucial for therapeutic decision making.

## Extra-myocardial finding

### FDG uptake in the coronary arteries

In atherosclerotic lesions, arterial inflammation indicates high risk for the progression of such as calcium deposition, plaque rupture, and plaque vulnerability, leading to future cardiovascular disease [129, 130]. FDG activity within plaques correlates with inflammation in atherosclerosis, especially for increased macrophage infiltration [131]. The higher levels of FDG uptake in the carotid arteries and the aorta increase the risk of cardiovascular disease [132, 133]. FDG accumulation was increased in the ascending aorta and left main coronary artery of patients who presented with acute coronary syndrome [134].

**Fig. 16:**
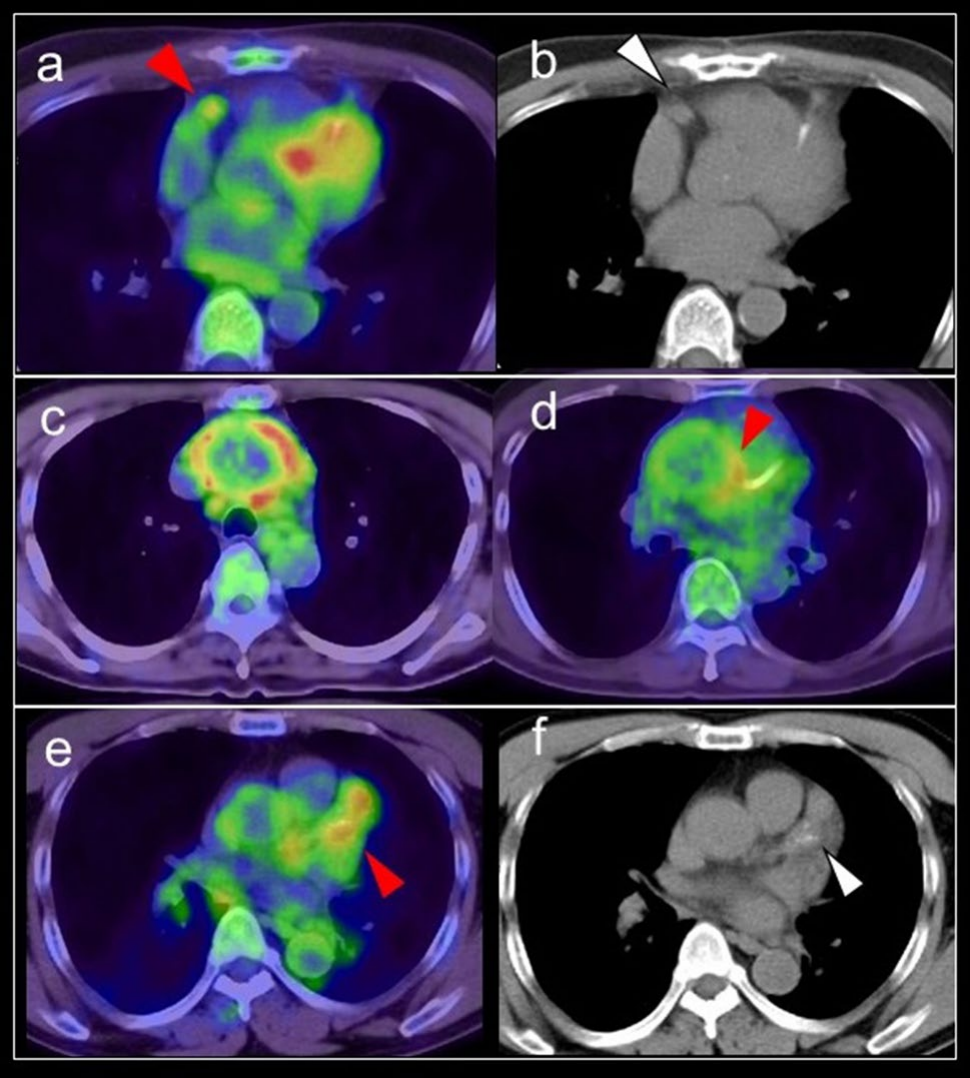
FDG uptake in coronary artery. Incidental findings of FDG uptake in the proximal of right coronary artery (**a** red arrowhead), suggesting the active plaque in the artery (**b** white arrowhead). The patient with Takayasu arthritis showed intense FDG uptake at ascending aorta (**c**), and the uptake reached the origin of right coronary artery (**d**). FDG uptake (**e** red arrowhead) in swollen left coronary artery (**f** white arrowhead) was diagnosed involvement of IgG4 related disease in coronary artery representing perivascular immuno-inflammation

In evaluation of FDG uptake in vulnerable plaque in the coronary arteries, patients follow a high-fat, low-carbohydrate diet prior to FDG-PET with the aim of minimizing myocardial glucose uptake to better visualize coronary FDG uptake [134, 135]. In standard FDG-PET/CT examination, as suppressed myocardial FDG uptake is not always expected, this finding would be confirmed occasionally. Moreover, because of the limited spatial resolution of PET imaging, partial volume effect, and motion, it remains challenging to identify FDG uptake in the coronary arteries. Although the proximal coronary arteries can be assessed by PET imaging [134], the mid and distal coronary vasculature is obscured by physiological uptake in the myocardium, and half of all coronary territories cannot be interpreted despite optimal myocardial suppression protocols [136–138]. Accordingly, high FDG uptake in the proximal coronary arteries may indicate active plaque causing cardiovascular disease, and can be encountered in a standard FDG-PET/CT scan (Fig. 16).

Coronary vasculitis is uncommon but can cause severe and life-threatening complications such as coronary artery aneurysm, coronary artery stenosis, intraluminal thrombosis, and microcirculation abnormalities. Takayasu arteritis (TAK), giant cell arthritis (GCA), polyarteritis nodosa, ANCA vasculitis, Erdheim-Chester disease, Kawasaki disease, Bechet’s disease, immunoglobulin G4 (IgG4) related periarteritis, are all associated with coronary vasculitis [139, 140]. The development of coronary artery stenosis in patients with TA is an extension of the inflammatory process and intimal proliferation in the ascending aorta. The incidence of coronary artery disease in TA has been reported as 10–45% in autopsy cases [141, 142]. FDG uptake in the coronary arteries was not confirmed in cases of TA accompanied by coronary ostial stenosis [143] (Fig. 16). Coronary artery involvement in IgG4 related disease can be confirmed as increased FDG uptake in the coronary arteries [144] (Fig. 16). This finding is caused by perivascular immuno-inflammation related to IgG4, which occurs in smaller vessels including the coronary arteries, as well as in large vessels [145, 146].

### FDG uptake in the pericardium

The underlying causes of uptake in the pericardium are neoplastic disease, infectious disease, and noninfectious disease. Considering of physiological FDG uptake in the myocardium, pericardial FDG uptake can be recognized in the specific condition as suppressed myocardial FDG uptake, very high and/or mass-like FDG uptake in the pericardium, uptake around the atrium, where FDG uptake is generally low, and the presence of some extent of pericardial effusion, which appears as a space between the myocardium and pericardium. Therefore, if pericardial abnormality is suspected, it is recommended that the patient preparation is designed to suppress myocardial physiological FDG uptake, by such as prolonged fasting and following a high-fat/low-carbohydrate diet [147]. Fever, subacute course, large effusion or tamponade, and aspirin or NSAID failure are specific clinical features identifying high risk for specific causal conditions and complications [148].

**Fig. 17:**
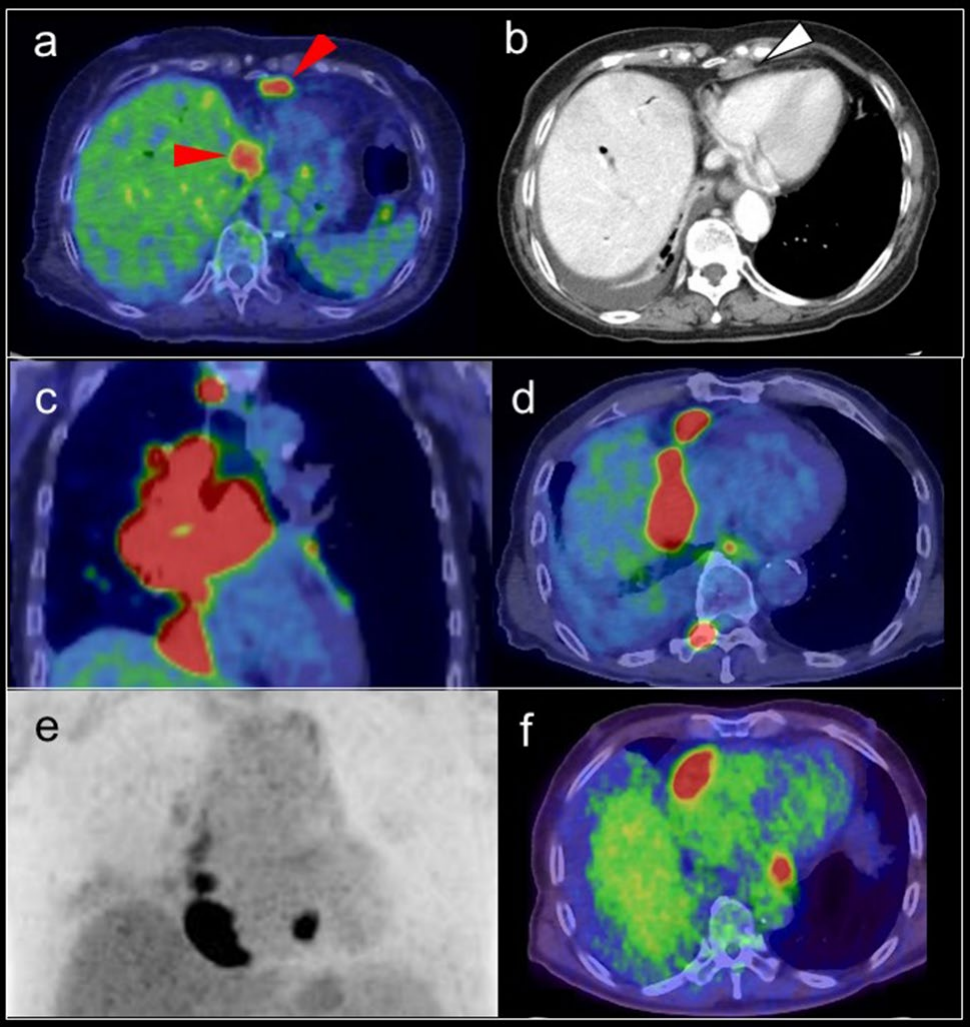
Pericardial lesions. FDG-PET/CT depicted pericardial metastatic lesions (red arrowhead) in patient with gastric cancer (**a**). Contrast enhanced CT showed just a lesion matched to lesion showing FDG uptake (**b**). Malignant lymphoma lesion was involved in the pericardium (**c**, **d**). Focal FDG uptake in the pericardium was proved pericardial invasion of tuberculosis

The most common malignancy of the pericardium is metastatic pericardial tumor, of which the primary lesion is most commonly from the lung, breast, or lymphoma. It is important to consider a previous history of malignancy in diagnosis (Fig. 17).

Primary tumor in the pericardium is rare, but includes mesothelioma, sarcoma, and lymphoma, which can be identified by FDG avidity in the pericardium [147]. The common feature of malignant disease of the pericardium is accompanying pericardial effusion, but this feature is also associated with generalized conditions such as hypoalbuminemia and anemia that occur in various types of progressive disease, including malignancy [149].

In patients who have no previous history of malignancy, acute pericarditis is caused by cancer pathogenesis in up to 5% of cases [148]. The incidence of malignancy increases to 23% when the pericarditis is accompanied by pericardial effusion [150, 151]. FDG-PET/CT can detect the primary lesion and tumor extension, and the identification of a pericardial lesion may result in changes to the more effective treatment plan.

Viral pericarditis is the most common and least severe type of acute pericarditis. It is usually self-limiting and rarely progresses to cardiac tamponade [148]. Clinically, viral pericarditis mimics idiopathic pericarditis in its manifestation. Although a mild disease in most cases, when combined with myocarditis there is a high risk of heart failure and the prognosis is poor [150]. Bacterial and fungal pericarditis is more common in immunocompromised patients and accompanied the infection in adjacent organs. A sensitivity of FDG-PET/CT is lower than echocardiography and CT for the diagnosis of pericarditis.

Tuberculous pericarditis has high morbidity and mortality and frequently results in constrictive pericarditis [152]. In addition to its value in evaluating pericarditis, FDG-PET/CT is useful for detecting extrapulmonary tuberculosis [153] (Fig. 17). The pericardial FDG uptake was characteristically diffuse or multifocal in acute tuberculous pericarditis and diffuse or regional in acute idiopathic pericarditis. The degree of FDG uptake in the pericardium and the mediastinal and supraclavicular lymph nodes is higher in acute tuberculous pericarditis than in idiopathic pericarditis [154].

The utility of SUV values for distinguishing between benign and malignant pericardial disease remains controversial, as the values vary broadly in both settings [155]. Zhang et al. reported that mean SUVmax was 1.75 (range 1.0–9.2) in a tumor group, which was significantly higher than that in a nontumor group (mean SUVmax, 1.1; range 0.7–2.2), but the differences were so small with a certain level of overlap.

Radiation-associated pericarditis can develop during or immediately after radiation therapy, but most typically presents around 1 year after the end of radiation treatment. Pericarditis is usually accompanied by asymptomatic pericardial effusion, with subsequent fibrosis in some patients [156]. The lower threshold of the radiation dose leading to radiation-associated heart disease is ~ 15 Gy, and cardiac complications increase at doses > 40 Gy [157]. Patients with radiation-induced pericarditis typically present with FDG uptake consistent with the radiation field; therefore, information in the radiation dose report can help distinguish between radiation-induced pericarditis and other pericardial disease [18].

**Fig. 18:**
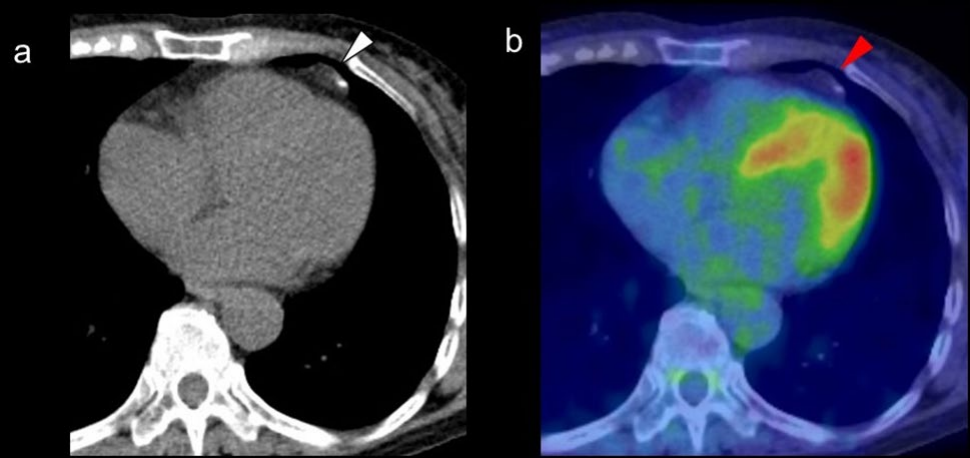
Epipericardial fat necrosis. CT showed small nodular lesion in the epipericardial fat (**a** white arrowhead), and it demonstrated low FDG uptake (**b** red arrowhead)

Epipericardial fat necrosis is a benign condition that commonly presents as acute pleuritic chest pain [158]. CT may show focal increased attenuation within the epipericardial fat [159], and it demonstrates low FDG uptake (Fig. 18).

### Possibility of systemic inflammatory disease

**Fig. 19:**
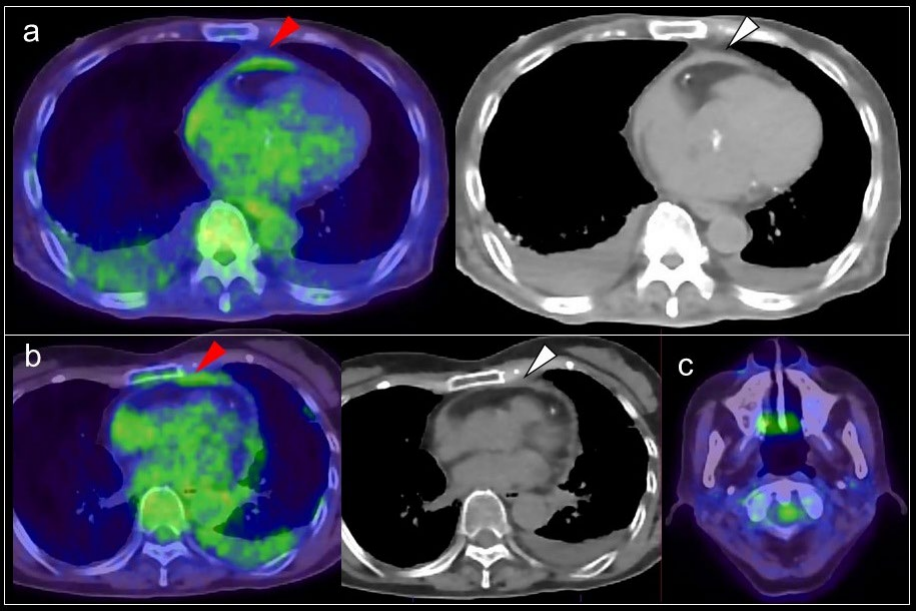
Pericardial lesion associated with systemic disease. **a** FDG uptake could be confirmed in the thickened pericardium, suggesting pericardium associated with SLE. **b** Moderate FDG uptake was seen in the part of thickened pericardium. It was acute pericarditis occurred in the patient was Sjögren syndrome showing the atrophic change in both parotid grands (**c**)

In systemic inflammatory disease, pericarditis can occur [160] in granulomatosis with polyangiitis (GPA) [161], eosinophilic granulomatosis with polyangiitis (EGPA) [162], systemic lupus erythematosus (SLE) [163], systemic sclerosis, mixed connective tissue disease, adult-onset still disease (AOSD) [164], and relapsing polychondritis (Fig. 19). GPA, EGPA, SLE (rare), and relapsing polychondritis can accompany cardiac artery disease; myocarditis may occur in EGPA, SLE, and AOSD; endocarditis can occur in GPA and SLE (Libman-Sacks endocarditis) [165]; cardiomyopathy in SLE; and valvular disease in SLE and relapsing polychondritis. Although the features of FDG uptake in the myocardium and pericardium have been reported for some types of systematic disease, it remains challenging to identify these systematic diseases by FDG-PET/CT imaging alone.

### CT portion of PET/CT

The CT portion of PET/CT can provide valuable information for identification of myocardial disease and/or other related disease. Lee et al. demonstrated incidental cardiac and pericardial abnormalities on chest CT, but it could be identified under the contrast media to be able to identify the hidden disease clearly [166]. The CT scanning during PET/CT examination is obtained with low dose radiation, without electrocardiogram gating and contrast media; thus identification of myocardial and pericardial abnormalities would be limited.

As the presence of coronary calcification is a risk factor for acute coronary events, its presence on CT scanning during PET/CT can identify individuals at risk for acute coronary events [167, 168]. Moreover, low-attenuation noncalcified plaque, positive remodeling, and small spottily distributed calcifications were reported to be associated with an increased likelihood of adverse events [169]. Intense FDG uptake in the coronary arteries indicates vascular inflammation and is associated with the progression of coronary artery calcification. However, sites of calcification on CT consistently demonstrate no or very minimal uptake of FDG, suggesting that inflammation is not a major component in the formation of atherosclerotic plaques [170–174]. A mild degree of calcification on CT is characteristic of acute coronary events, whereas diffused high-attenuation calcific plaques are related to chronic coronary events [175].

**Fig. 20:**
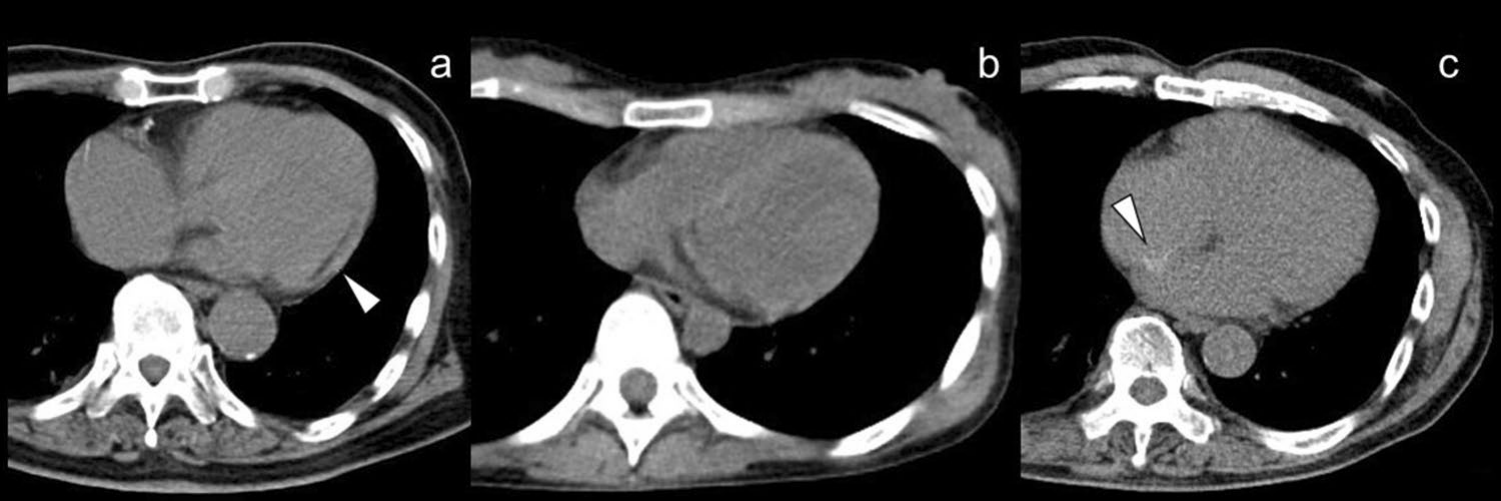
Incidental CT findings in FDG-PET/CT. A curvilinear dark line (white arrowhead) in the myocardium indicating fibrosis in patients with a history of myocardial infarction (**a**). The interventricular septum was visualized in patient with low hemoglobin concentration due to the decreased CT density in blood pool (**b**). The patient had a history of occluding interatrial septal defect (**c**). A high-density foci (white arrowhead) in the interatrial septal indicated the operative site

A curvilinear dark line in the myocardium indicating fibrosis or fatty metaplasia is seen in 22–62% of patients with a history of myocardial infarction [176] (Fig. 20). This finding is confirmed also in LV aneurysms that developed from myocardial infarction [177]. Other entities that show abnormal myocardial fat include arrhythmogenic right ventricular cardiomyopathy or dysplasia (ARVC), cardiac lipoma, LHIAS, tuberous sclerosis, complex tuberculosis, dilated cardiomyopathy, and muscular dystrophy [176].

Myocardial calcification is a reliable sign of previous myocardial infarction. Septal involvement and regional myocardial thinning help to differentiate these lesions from those of calcific pericarditis [166].

The possibility of congenial heart disease and heart failure can be implied by irregularly shaped myocardium (Fig. 20). Aortic stenosis may be suspected by aortic valve calcification with LV and aortic dilatation or LV hypertrophy supported by FDG uptake features. Mitral annulus calcification is a degenerative process of the fibrous support structure of the mitral valve. It may not affect the function of mitral valve, but it is an additional marker of atherosclerosis [178].

Visualization of the interventricular septum suggests abnormally low hemoglobin concentration, in patients with glycogen and iron storage diseases and also in patients with iron overload caused by multiple blood transfusions in the presence of normal hemoglobin levels [179] (Fig. 20). On thoracic CT, a CT density threshold of 35 HU has been used to differentiate between anemic and nonanemic states [180, 181].

## Conclusion

Due to the variety of physiological FDG uptake in the myocardium, the interpretation of cardiac FDG uptake in standard FDG-PET/CT is usually uncertain. However, recognition of possible underlying disease based on knowledge of common patterns of myocardial FDG uptake will support further patient management to avoid complications due to the disease.
